# Clinical outcomes of persistent cough following coronavirus disease 2019 infection: A 1-year retrospective cohort study

**DOI:** 10.5415/apallergy.0000000000000188

**Published:** 2025-03-17

**Authors:** Sang Pyo Lee, Sung-Yoon Kang

**Affiliations:** 1 Division of Pulmonology and Allergy, Department of Internal Medicine, Gachon University Gil Medical Center, Incheon, Republic of Korea.

**Keywords:** Adults, coronavirus disease 2019, long COVID, persistent cough

## Abstract

**Background:**

Cough is one of the multiple prolonged symptoms observed in patients who had coronavirus disease 2019 (COVID-19) infection.

**Objective:**

We assessed the clinical outcomes and identified factors contributing to cough persistence in patients post-COVID-19.

**Methods:**

This retrospective cohort study included adults who visited a specialist cough clinic between 2022 and 2023. All participants underwent systematic investigation and treatment for persistent cough. Cough persistence was assessed at the 2- and 12-month follow-ups. Participants were classified as having persistent cough if they had a current troublesome cough at the 2- and 12-month follow-ups, and a cough severity visual analog scale (VAS) score change below 30.

**Results:**

Sixty-six patients (mean age 48.7 years; 72.7% women) were analyzed and divided into 2 groups: persistent cough (33.3%) and remitted cough (66.7%). The persistent cough group had a significantly higher prevalence of abnormal laryngeal sensation, sputum production, breathing difficulty, and airway eosinophilia; their VAS score changes at 2 months were also lower. Multivariable analyses indicated associations between persistent cough at 1 year and factors such as airway eosinophilia (adjusted odds ratio [aOR], 6.78), abnormal laryngeal sensation (aOR, 6.42), and low cough VAS reduction (aOR, 1.05).

**Conclusion:**

Persistent cough remained a significant issue for one-third of the patients after COVID-19. The clinical features commonly observed in chronic cough were also present in those who have experienced COVID-19, which contributed to prolonged cough. These findings underscore the need for systematic assessment and tailored treatment strategies to effectively manage persistent cough in patients post-COVID-19.

## 1. Introduction

The coronavirus disease 2019 (COVID-19) pandemic, which began in 2019 and has continued to significantly affect the world through 2023, has resulted in substantial global morbidity and mortality, altering daily lives, economies, and social structures worldwide [1–3]. The stringent measures implemented to curb the spread of the virus, including lockdowns, travel restrictions, and social distancing, led to widespread economic and social disruptions. The impact of COVID-19 has extended far beyond the acute phase of infection. Increasing evidence suggests that COVID-19 often leaves behind a range of lingering aftereffects, collectively referred to as “long COVID” or post-COVID-19 syndrome [4, 5]. This syndrome encompasses a wide array of symptoms that can persist or even emerge well after the resolution of the acute infection.

Among the various symptoms associated with COVID-19, cough has emerged as a particularly concerning issue due to its persistence in a considerable subset of patients [6, 7]. Although cough is a well-known symptom during the COVID-19 acute phase, its persistence as a chronic condition long after recovery raises important clinical concerns. Several studies have explored the immediate respiratory effects of COVID-19, particularly during this acute phase [8–12]. However, there remains a substantial gap in the literature concerning the long-term respiratory impacts, particularly regarding cough.

Given the wide-ranging and potentially debilitating nature of long COVID-19 symptoms, it is imperative to conduct comprehensive studies on the long-term effects of COVID-19 [13, 14]. Therefore, this study was designed to systematically investigate 2 primary objectives in outpatient subjects: (1) the prevalence of persistent cough among patients who have recovered from COVID-19 and (2) the clinical and demographic factors that may contribute to the persistent cough. By addressing these objectives, we aimed to fill the existing knowledge gaps and provide valuable insights that could inform both clinical practice and future research efforts in managing patients post-COVID-19.

## 2. Methods

This retrospective cohort study was conducted with adults who visited a specialist cough clinic at a tertiary hospital in Incheon, Republic of Korea, between 2022 and 2023. These patients had confirmed diagnoses of COVID-19 diagnoses (via positive polymerase chain reaction or antigen tests for severe acute respiratory syndrome coronavirus 2) and were experiencing post-COVID persistent cough lasting >8 weeks after their initial recovery from COVID-19. All participants reported being completely healthy before the COVID-19 infection, without a long-term respiratory disease diagnosis or treatment. Although the optimal assessment and management of COVID-19-associated cough remain unclear, all patients in this study were evaluated using anatomical diagnostic protocols and treated in accordance with the guidelines for chronic cough [15]. Patients were also asked to complete a structured questionnaire that gathered information on demographic characteristics, COVID-19-related symptoms beyond cough, and cough severity using tools such as the 100-mm linear visual analog scale (VAS), Cough Symptom Score, and Leicester Cough Questionnaire (LCQ) to assess cough-related quality of life [16, 17]. Follow-ups were conducted at 2- and 12-months to evaluate cough persistence. Face-to-face interviews were conducted for patients who regularly attended the clinic, while telephone interviews were conducted for those who did not visit the clinic during their scheduled follow-up period. To minimize recall bias, both types of interviews were conducted at the same follow-up time points. All interviews were carried out by an experienced research nurse to ensure clear communication and consistency in the information provided by patients. Additionally, a structured questionnaire was used to provide all patients with identical questions. At the 2-month follow-up, the proportion of patients interviewed face-to-face and by telephone was 71% and 29%, respectively, whereas at the 1-year follow-up, these proportions were 39% and 61%.

Patients were classified as having persistent cough if they continued to experience troublesome cough at both the 2- and 12-month follow-ups, with changes in cough severity on the VAS remaining below the minimum clinically important difference (MCID) threshold despite treatment. Remitted cough was defined as a condition in which patients, at both follow-up periods, either had no cough without treatment or demonstrated an improvement exceeding the MCID threshold with treatment. According to Martin Nguyen et al. [18], the MCID for the cough VAS score was set at 30.

Ethical approval for this study (IRB No. GBIRB2022-259) was provided by the Ethics Committee of Gachon University Gil Medical Center, Incheon, Republic of Korea (Chairperson Prof. Byung Joon Kim) on September 15, 2022. The requirement for informed consent was waived due to the retrospective nature of the study.

### 2.1. Statistical analysis

Differences between the groups (persistent cough vs remitted cough) were evaluated using Student’s *t* test for continuous variables and the chi-square test for categorical variables. Univariate and multivariable logistic regression analyses were conducted to explore factors for persistent cough versus remitted cough. All statistical analyses were performed using SPSS version 27.0 (IBM Corp., Armonk, NY, USA). Two-sided *P* values <0.05 were considered statistically significant.

## 3. Results

A total of 66 participants were included in the study, of whom 22 (33.3%) had persistent cough and 44 (66.7%) had remitted cough following COVID-19 infection. The baseline characteristics of the study population are summarized in **Table 1**. The study participants had a mean age of 48.7 years, a mean body mass index of 25.0 kg/m^2^, and were mostly women (72.2%) and nonsmokers (69.6%). The mean duration of cough was 140.56 days, and the onset of cough was 7 days after COVID-19. The most common comorbidities were dyslipidemia (18.2%) and hypertension (15.2%). Approximately half of the participants reported new-onset persistent symptoms other than cough, such as neurologic symptoms (34.4%), ear, nose, and throat symptoms (31.3%), neuropsychiatric symptoms (18.8%), and general symptoms (15.6%).

**Table 1.** Demographics and clinical characteristics between the persistent and remitted cough groups in 66 patients with COVID-19

Characteristics	Total (N = 66)	Remitted cough (N = 44)	Persistent cough (N = 22)	*P* value
Sex, n (%)				
Male	18 (27.3)	10 (22.7)	8 (36.4)	0.241
Female	48 (72.7)	34 (77.3)	14 (63.6)	
Age (years)	48.70 ± 15.57	48.18 ± 17.22	49.73 ± 11.87	0.707
Age group, n (%)				0.736
19–29	10 (15.2)	9 (20.5)	1 (4.5)	
30–39	9 (13.6)	5 (11.4)	4 (18.2)	
40–49	14 (21.2)	8 (18.2)	6 (27.3)	
50–59	12 (18.2)	8 (18.2)	4 (18.2)	
60–69	16 (24.2)	9 (20.5)	7 (31.8)	
70 or more	5 (7.6)	5 (11.4)	0 (0.0)	
Smoking status, n (%)				0.295
Nonsmoker	46 (69.6)	32 (72.7)	14 (63.6)	
Ex-smoker	10 (15.2)	7 (15.9)	3 (13.6)	
Smoker	10 (15.2)	5 (11.4)	5 (22.7)	
Body mass index (kg/m²)	25.00 ± 5.02	24.86 ± 5.49	25.27 ± 4.03	0.755
Comorbidities, n (%)				
Hypertension	10 (15.2)	6 (13.6)	4 (18.2)	0.720
Diabetes mellitus	4 (6.1)	3 (6.8)	1 (4.5)	1.000
Cardiovascular disease	2 (3.0)	2 (4.5)	0 (0.0)	0.549
Dyslipidemia	12 (18.2)	7 (15.9)	5 (22.7)	0.515
Gastroesophageal reflux disease	3 (4.5)	2 (4.5)	1 (4.5)	1.000
Thyroid disease	7 (10.6)	4 (9.1)	3 (13.6)	0.678
Family history of chronic cough, n (%)	4 (6.1)	2 (4.5)	2 (9.1)	0.596
Family history of allergy, n (%)	14 (21.2)	8 (18.2)	6 (27.3)	0.524
Cough interval after COVID-19, days	7.80 ± 15.57	9.25 ± 19.03	4.91 ± 6.35	0.176
Cough duration, days	140.56 ± 83.56	142.34 ± 85.69	137.00 ± 80.97	0.809
Abnormal laryngeal sensations, n (%)	15 (22.7)	6 (13.6)	9 (40.9)	0.013
Sputum, n (%)	31 (41.7)	16 (36.4)	15 (68.2)	0.015
Dyspnea, n (%)	38 (57.6)	20 (45.5)	18 (81.8)	0.005
Wheezing, n (%)	15 (22.7)	6 (13.6)	9 (40.9)	0.013
Nasal symptoms, n (%)	35 (53.0)	21 (47.7)	14 (63.6)	0.222
Reflux/Soreness, n (%)	27 (40.9)	18 (40.9)	9 (40.9)	1.000
Visual analog scale (VAS)	68.79 ± 23.83	70.91 ± 23.61	64.55 ± 24.25	0.310
VAS change at 2 months	46.97 ± 31.18	55.45 ± 26.80	30.00 ± 32.95	0.001
VAS score at 1 year	24.55 ± 32.87	6.14 ± 13.16	61.36 ± 29.33	<0.001
Cough symptom score, total	5.09 ± 1.67	5.09 ± 1.68	5.09 ± 1.69	1.000
Cough symptom score at day	2.95 ± 1.03	3.00 ± 1.03	2.86 ± 1.04	0.616
Cough symptom score at night	2.14 ± 1.14	2.09 ± 1.05	2.23 ± 1.31	0.649
Leicester Cough Questionnaire, total	12.44 ± 4.51	12.48 ± 4.45	12.36 ± 4.74	0.917
Physical	4.25 ± 1.35	4.24 ± 1.35	4.25 ± 1.38	0.987
Psycho	3.82 ± 1.54	3.86 ± 1.50	3.73± 1.66	0.750
Social	4.38 ± 1.82	4.38 ± 1.81	4.38 ± 1.86	1.000
Other symptoms after COVID-19, n (%)*	32 (48.5)	19 (43.2)	13 (59.1)	0.223
Neurologic symptoms	11 (34.4)	7 (36.8)	4 (30.8)	
Ear, nose, and throat symptoms	10 (31.3)	6 (31.6)	4 (30.8)	
Neuropsychiatric symptoms	6 (18.8)	3 (15.8)	3 (23.1)	
General symptoms	5 (15.6)	3 (15.8)	2 (15.4)	
Dermatologic symptoms	4 (12.5)	2 (10.5)	2 (15.4)	
Cardiopulmonary symptoms	3 (9.4)	2 (10.5)	1 (7.7)	
Gastrointestinal symptoms	1 (3.1)	1 (5.3)	0 (0.0)	
Airway eosinophilia†	28 (42.4)	13 (29.5)	15 (68.2)	0.003
WBC (cells/uL)	7225.30 ± 2246.70	7581.82 ± 2325.61	6512.27 ± 1936.58	0.068
Neutrophil%	58.11 ± 11.12	59.65 ± 11.94	55.05 ± 8.75	0.114
Eosinophil%	3.93 ± 3.17	3.46 ± 3.03	4.87 ± 3.31	0.089
Erythrocyte sedimentation rate (mm/hr)	11.09 ± 9.33	11.32 ± 9.85	10.64 ± 8.40	0.782
Total IgE levels (KU/L)	200.78 ± 333.22	169.93 ± 254.55	262.49 ± 452.78	0.291
Atopy, n (%)‡	14 (21.2)	11 (25.0)	3 (13.6)	0.354
FEV_1_, Liters (L)	2.70 ± 0.62	2.68 ± 0.61	2.75 ± 0.66	0.701
FEV_1_%	90.41 ± 12.07	92.27 ± 10.01	86.68 ± 14.96	0.076
FVC, L	3.45 ± 0.76	3.40 ± 0.77	3.56 ± 0.74	0.401
FVC%	92.20 ± 10.95	93.23 ± 10.17	90.14 ± 12.35	0.283
FEV_1_/FVC%	79.06 ± 8.11	80.00 ± 7.03	77.18 ± 9.85	0.185
FEF_25-75%_ L/sec	2.63 ± 1.03	2.67 ± 0.99	2.58 ± 1.12	0.745
FEF_25-75%_ %	86.83 ± 25.42	88.82 ± 24.14	82.86 ± 27.97	0.374
RV/TLC%	29.95 ± 7.72	29.61 ± 8.50	30.64 ± 6.00	0.616
DLCO%	87.26 ± 14.48	88.11 ± 15.43	85.55 ± 12.51	0.501
Fractional exhaled nitric oxide (ppb)	41.30 ± 36.54	34.16 ± 26.79	55.59 ± 48.41	0.063
FEV_1_/FVC <70%, n (%)	8 (12.1)	5 (11.4)	3 (13.6)	1.000
Positive bronchodilator response, n (%)	2 (3.0)	1 (2.3)	1 (4.5)	1.000
Positive methacholine bronchial provocation test, n (%)	6 (9.1)	3 (6.8)	3 (13.6)	0.392
PNS abnormality, n (%)	43 (65.2)	29 (65.9)	14 (63.6)	0.855
CXR abnormality, n (%)	4 (6.1)	2 (4.5)	2 (9.1)	0.596
CT abnormality, n (%)	24 (36.4)	15 (34.1)	9 (40.9)	0.587

CT, computed tomography; CXR, chest X-ray; DL_CO_, carbon monoxide diffusion capacity; FEF_25-75%_, forced mid-expiratory flow; FEV_1_, forced expiratory volume in 1 s; FVC, forced vital capacity; PNS, paranasal Sinuses; RV, residual volume; TLC, total lung capacity; WBC, white blood cell.

* Neurologic symptoms category included headache, sleep disturbance, and myalgia. Ear, nose, and throat symptoms category included hyposmia and hypogeusia. Neuropsychiatric symptoms category included decreased attention. General symptoms category included fatigue, generalized weakness, and weight loss. Dermatologic symptoms category included hair loss and skin rash. Cardiopulmonary symptoms category included chest pain. Gastrointestinal symptoms category included abdominal discomfort.

† Airway eosinophilia was defined as high FeNO (≥25 ppb) or blood eosinophil (≥300 cells/µL).

‡ Atopy was defined as a positive response of specific IgE blood tests and multiple allergen simultaneous test (MAST) to at least one allergen with a rating of class 3 or more.

Baseline cough severity, including VAS, Cough Symptom Score, and LCQ were similar between the 2 groups, although the participants with persistent cough were more likely to have abnormal laryngeal sensations (40.9%), sputum production (68.2%), dyspnea (81.8%), and wheezing (40.9%) than those with remitted cough. Airway eosinophilia, defined as high fractional exhaled nitric oxide (FeNO) (≥25 ppb) or blood eosinophil (≥300 cells/µL), was significantly higher in the persistent cough group (68.2%). There were no significant differences in the family history of cough, comorbidities, cough interval and duration following COVID-19 infection, presence of symptoms other than cough, or diagnostic test results, including chest radiography, spirometry, atopy, and complete blood count profiles.

**Figure 1.**
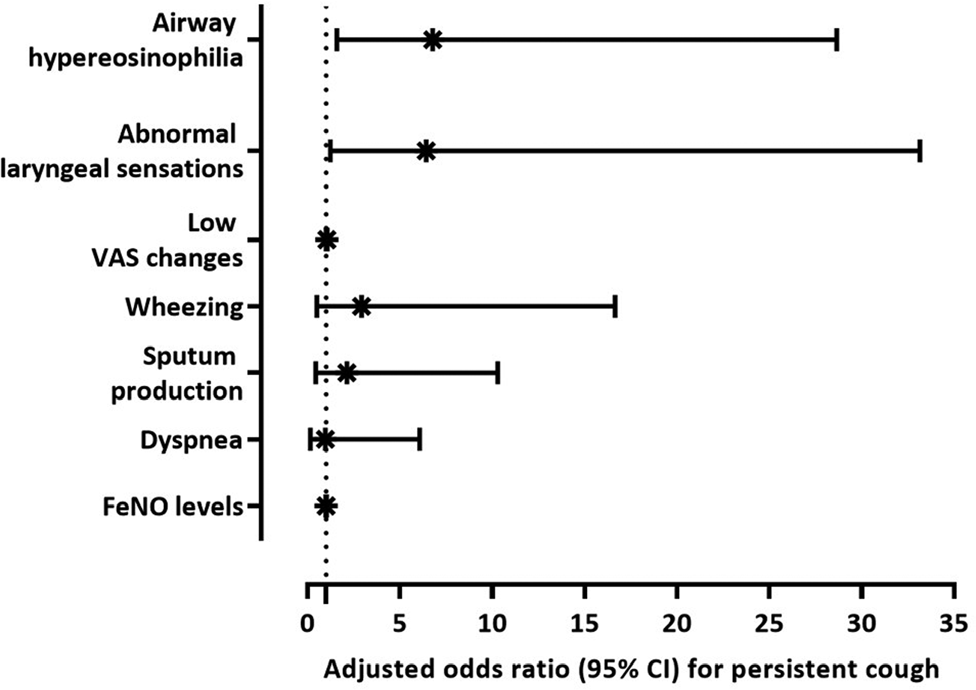
Multivariable logistic regression analysis of persistent cough at the 12-month follow-up. Multivariable logistic regression was performed to identify factors associated with persistent cough, adjusting for sex (female), age, smoking history, and baseline LCQ score. The forest plot presents the adjusted odds ratios and corresponding 95% confidence intervals. FeNO, fractional exhaled nitric oxide; LCQ, Leicester Cough Questionnaire; VAS, visual analog scale.

Univariate and multivariable logistic regression analyses were conducted to identify the potential predictors of persistent cough after COVID-19 infection. In univariate analyses, airway eosinophilia (odds ratio [OR], 5.11; 95% confidence interval [CI], 1.69–15.45; *P* = 0.003), abnormal laryngeal sensation (OR, 4.39; 95% CI, 1.31–14.70; *P* = 0.013), low cough VAS reduction (OR, 1.03; 95% CI, 1.01–1.06; *P* = 0.004), sputum production (OR, 3.75; 95% CI, 1.26–11.12; *P* = 0.015), dyspnea (OR, 5.40; 95% CI, 1.57–18.57; *P* = 0.005), wheezing (OR, 4.39; 95% CI, 1.31–14.70; *P* = 0.013), and FeNO level (OR, 1.02; 95% CI, 1.00–1.03; *P* = 0.039) (Supplementary Table S1, http://links.lww.com/PA9/A58). In the multivariable logistic regression analyses, we found significant associations between a persistent cough and the parameters airway eosinophilia (adjusted odds ratio [aOR], 6.78; 95% CI, 1.60–28.66; *P* = 0.009), abnormal laryngeal sensation (aOR, 6.42; 95% CI, 1.24–33.15; *P* = 0.026), and low cough VAS reduction (aOR, 1.05; 95% CI, 1.02–1.08; *P* = 0.001) (Fig. 1). These associations remained significant even after adjusting for baseline LCQ scores and demographic factors such as age, sex, and smoking status.

## 4. Discussion

This retrospective cohort study found that one-third of the participants had a persistent cough for approximately 1 year after COVID-19 infection determined by systematic assessment and management at a tertiary cough clinic. Several cough characteristics, such as airway eosinophilia, and low changes in symptom scores and cough-related laryngeal sensations were positively associated with cough persistence. These findings suggest that specific cough characteristics may predict the natural course of post-COVID-19 cough that requires targeted diagnostic and therapeutic approaches. Several studies have identified factors associated with cough post-COVID-19 [19, 20]. Watase *et al.* [19] conducted a longitudinal questionnaire survey on cough over a 12-month period with 690 patients who were infected with COVID-19. Cough prevalence decreased significantly by 4.4% from the acute phase to the 12-month follow-up. At the 12-month follow-up, they identified that COVID-19 severity, including ventilator management, correlated with prolonged cough and sputum production. In a cross-sectional study by Kanemitsu *et al.* [20] the sequelae of COVID-19 in 170 patients admitted to hospital was investigated [20]. Cough was prevalent in 24% of the participants after recovering from COVID-19 and often accompanied by other symptoms, particularly gastrointestinal symptoms, which were found to have a strong correlation with cough presence before contracting COVID-19 and the presence of sputum and abnormal laryngeal sensations (measured by the Newcastle Laryngeal Hypersensitivity Questionnaire); these were also significant contributors to chronic cough and its severity.

We found that cough persisted in approximately 33% of patients with cough after 1 year of observation following systematic assessment and treatment, which was higher than the persistence rates reported in these 2 studies. Those study findings are not comparable because the patient characteristics and endpoint definitions were different. However, the authors suggest that cough may persist as a long-term symptom in a considerable proportion of patients who were infected with COVID-19. Neuroviral interactions in COVID-19 may be associated with increased cough reflex sensitivity during the COVID-19 acute phase [21–23]. Comparing the findings of this study with other studies, clinical indices during the COVID-19 acute phase were not associated with persistent cough development, suggesting that the pathophysiology of cough after recovery is distinct from that of the COVID-19 acute phase, and more research is required to elucidate the mechanisms of chronic cough development from COVID-19. Furthermore, these findings emphasize that coughs are not merely acute sequelae; they may represent an ongoing burden on patients after recovery from acute COVID-19, indicating the importance of monitoring and managing cough effectively.

The predictors of cough persistence in patients who were infected with COVID-19 are largely unknown. There is a lack of studies on the predictors of persistent cough after COVID-19, particularly for relatively healthy individuals without a history of respiratory tract diseases. In 2 cohort studies in Japan, continuous cough was significantly associated with the presence of severe COVID-19, gastrointestinal symptoms, sputum, and chronic cough before COVID-19 [19, 20]. Our study revealed that several cough characteristics, such as airway eosinophilia, abnormal laryngeal sensation, and low symptom improvement after a 2-month treatment period, were potential predictors of cough persistence. Airway eosinophilia, which encompasses various classifications such as classic asthma, cough-variant asthma, and nonasthmatic eosinophilic bronchitis, is responsive to inhaled corticosteroid (ICS) treatment. In this study, patients with high levels of FeNO or peripheral blood eosinophil counts—both established as reliable markers of airway eosinophilia—were treated with at least a medium dose of inhaled corticosteroid [15, 24].

In a recent study on Korean patients, persistent cough characteristics were similar to those observed in patients without COVID chronic cough [25]. The FeNO levels were significantly higher in patients with post-COVID-19 cough than in those without COVID-19 chronic cough. This suggests that T2 inflammation, possibly due to the viral exacerbation of subclinical eosinophilic bronchitis or asthma, may play a crucial role in cough persistence after COVID-19. Airway eosinophilia, as a treatable trait, has significant implications for respiratory condition management, particularly post-COVID-19 persistent cough. Identifying airway eosinophilia in post-COVID-19 cough can lead to more effective management by addressing the underlying inflammation that contributes to coughing. This approach not only improves patient outcomes but also minimizes the cough burden and enhances quality of life.

Abnormal laryngeal sensations, including tickling, a sensation of a lump in the throat, irritation, dryness, soreness, and throat mucus, are frequently observed in patients with chronic cough, especially in those with refractory or unexplained chronic cough [26, 27]. Although a Japanese study measured abnormal laryngeal sensation using a validated questionnaire tool, our findings should be carefully interpreted because the abnormal laryngeal sensation was merely evaluated by the questionnaire and not by objective tests. However, these findings beyond the acute phase in a subset of patients suggest an association between neuroviral interactions and abnormal laryngeal sensations, which warrants further investigation.

Despite the high negative impact of post-COVID-19 syndrome, clinical evidence and guideline recommendations for its management are still lacking. In a Korean study, most patients with post-COVID-19 cough responded well to cough guideline-based usual care [25]. However, some patients did not show improvement and were refractory to treatment. Those findings underscore that persistent cough after COVID-19 is a heterogeneous syndrome, much like usual chronic cough, and may require a differential diagnosis to identify other underlying causes despite usual care. Further studies are required to investigate and improve the outcomes of patients with persistent cough after COVID-19.

Our study has certain limitations that must be addressed. First, it relied on subjective self-reported measures and did not evaluate objective cough measures. The definition of persistent cough after COVID-19 is arbitrary, which has contributed to a lack of clarity regarding the nature of the cough. Second, face-to-face interviews were conducted with patients who regularly attended the clinic or via telephone with those who did not regularly visit the clinic. Telephone interviews are shorter and less comprehensive than face-to-face interviews, and the quantity and quality of data may have been compromised. Third, different COVID-19 viral strains have led to varying clinical outcomes [28–30]. The recruitment period coincided with the predominance of the Omicron variant in the Republic of Korea, and it is not certain whether these features are specific to Omicron among various variants. The Delta variant, which became dominant in many parts of the world by mid-2021, is linked to more severe disease and higher hospitalization rates. Omicron is generally associated with milder disease and demonstrates a tendency to affect the upper respiratory tract more than the lower respiratory tract, which could explain the lower incidence of severe pneumonia and respiratory failure observed with Omicron than with Delta. This highlights the importance of future research to investigate the influence of various viral strains on the development and persistence of cough. Fourth, we found several characteristics that were significantly associated with cough persistence at 1 year; however, their causal relationships warrant confirmation in prospective longitudinal cohort studies. Finally, our study population consisted of patients referred to a tertiary hospital cough outpatient clinic for persistent cough following COVID-19. Consequently, the findings may not fully represent the entire spectrum of COVID-19 patients, ranging from those who recover spontaneously or improve with primary care to those experiencing severe cough requiring admission. This selection bias limits the generalizability of our results. To address this limitation, future studies should include a more diverse patient population and adopt a multicenter design to better capture the full range of post-COVID-19 chronic cough cases. Despite these limitations, the strength of our study lies in its focus on a relatively healthy population, which allowed for a clear observation of the natural course of cough after COVID-19. To clearly identify the impact of COVID-19 on persistent cough, we analyzed a population that was not influenced by confounding factors, such as additional respiratory infections or other triggers, during the follow-up period. Moreover, the study design facilitated the identification of factors associated with a persistent cough, offering valuable insights into the assessment and management of post-COVID-19 persistent cough.

In conclusion, the cough persisted in one-third of our patients after 1 year of observation following the COVID-19 infection. Several cough characteristics, such as airway eosinophilia, abnormal laryngeal sensations, and delayed symptom resolution, may be associated with a persistent cough. Cough is the most common respiratory symptom of acute COVID-19 and can continue to burden patients even after recovery, making it crucial for physicians to focus on thorough assessment and meticulous management of post-COVID-19 persistent cough. Further studies are required to confirm these associations and explore their causal relationships.

## Conflicts of interest

The authors have no financial conflicts of interest.

## Acknowledgments

The authors would like to thank all the study participants. We are especially grateful to the research coordinators Min-Seo Kim, Seolyeon Kyung, and Hae-Rim Kwon for their assistance in patient interviews and data extraction. This study was supported by the Gachon University Gil Medical Center (FRD2021-13).

## Supplementary material

Supplementary Table S1 can be found via 10.5415/apallergy.2022.12.e38

Supplementary Table S1

Click here to view
